# Forecasting the number of sunspots for solar cycle 25 utilizing the facebook prophet model

**DOI:** 10.1038/s41598-025-33819-5

**Published:** 2026-01-15

**Authors:** H. I. Abdel Rahman, W. A. Badawy

**Affiliations:** Astronomy Department, National Research Institute of Astronomy and Geophysics, Helwan, Cairo, 11421 Egypt

**Keywords:** Solar cycle, Time series analysis, Sunspots, Statistical model, FB Prophet Prediction Model., Astronomy and planetary science, Climate sciences, Energy science and technology, Space physics

## Abstract

The solar cycle, also referred to as the solar magnetic activity cycle, represents a nearly periodic change in solar activity occurring approximately every 11 years, as evidenced by the observation of sunspot numbers. The terms solar maximum and minimum denote the phases of peak and trough sunspot activity, respectively. Solar Cycle 25 commenced in December 2019, starting with a minimum smooth sunspot number of 1.8, and is projected to persist until the conclusion of December 2030. In this study, we employed the FB Prophet Prediction Model, utilizing sunspot data collected from January 1749 to March 2025 (spanning over 276.25 years), to forecast sunspot numbers for the latter half of Solar Cycle 25 (69 months). We forecast sunspot numbers for the remainder of Solar Cycle 25 and the entirety of Solar Cycle 26 (through 2036). This study employed the FB Prophet model on 276 years of sunspot data (January 1749–March 2025) to generate two forecasts: one for the remainder of Solar Cycle 25, and a second for the early portion of Solar Cycle 26, extending through 2036. Our model predicts that Solar Cycle 25 will peak in early 2025 and that Solar Cycle 26 will peak in mid-2034, both with a smoothed sunspot number of approximately 118. A comparison between our predicted outcomes and the NOAA published forecast data demonstrates the effectiveness and suitability of the FB Prophet Prediction model for predicting sunspot activity during Cycle 25. The coefficient of determination, commonly referred to as ($R^{2}$), assesses the extent to which the model reflects the observed outcomes and signifies the percentage of variance in the dependent variable that can be forecasted based on the independent variables within the model. Its value is 89.23%, which demonstrates the model’s high level of predictive accuracy. This demonstrates the good agreement results and also confirms the effectiveness and suitability of the FB Prophet Prediction model for predicting sunspot activity during Cycle 25.

## Introduction

The Sun has commenced its 25th solar cycle and is poised for resurgence. In recent years, our sun has exhibited a period of relative tranquility, characterized by a scarcity of sunspots, luminous flares, and significant ejections of magnetized plasma from its surface. This phase of diminished activity is referred to as the solar minimum; however, indications suggest that activity is set to escalate once again. Numerous researchers have employed various methodologies to project sunspot number for forthcoming solar cycles^1–4^. In their contemporary approach to solar cycle forecasting, they utilized two methods of nonlinear least squares fitting to analyze annual sunspot data^5^, resulting in a forecast for the impending solar cycle. Additionally, Abdel Rahman and Marzouk^6^ implemented a different model based on Box-Jenkins techniques to estimate the sunspot number for cycle 24 and Abdel Rahman et al. used ARIMA model to predict the sunspot number for solar cycle 25.

Various forecasts regarding the intensity of Cycle 25 have been suggested, with some indicating a notably weak cycle that may lead to a state reminiscent of the Maunder Minimum^7^, while others predict a weak cycle akin to the preceding Cycle 24, and there are also expectations of a robust cycle.

Upton and Hathaway^7^ forecasted that the diminished activity of cycle 25 would categorize it within the Modern Gleissberg Minimum. The Solar Cycle 25 Prediction Panel, in December 2019^8^, anticipated that solar cycle 25 would resemble solar cycle 24, with the preceding solar cycle minimum occurring in April 2020 ($\pm 6$ months), and the number of sunspots expected to reach a (smoothed) peak of 115 in July 2025 ($\pm 8$ months).

Solar cycle prediction methodologies are broadly categorized into precursor methods, which use indicators like polar magnetic fields; physical models, such as flux transport dynamos; and statistical/machine learning approaches that extrapolate historical patterns^9,10^. Forecasts for Solar Cycle 25 have varied, with some models predicting a weak cycle similar to Cycle 24 and others forecasting a significantly stronger one^11^. Recent works have utilized diverse techniques, from surface flux transport^12^ to machine learning applied to historical data^13^, highlighting the ongoing challenge and importance of multi-model comparisons.

Of much greater interest would be a forecast of the SSN index for one full 11-year cycle ahead, up to the maximum of Cycle 26. Such extended forecasts are already being made by several researchers using various methodologies. For instance, Pei-Xin Luo and Bao-Lin Tan employed a modified logistic growth model to predict Solar Cycle 25 and provided an early outlook for Cycle 26, highlighting the challenges in long-term solar activity forecasting^14^. Similarly, Zeng et al. utilized a deep learning framework incorporating polar field observations to project the amplitude and timing of Cycle 26, demonstrating the potential of machine learning in solar cycle prediction^15^. Abdulkadir et al. applied a hybrid model combining wavelet transforms and neural networks to forecast sunspot numbers through Cycle 26, emphasizing the importance of capturing both short-term fluctuations and long-term cyclical patterns^16^. These studies represent the growing interest in extending solar cycle predictions beyond the immediate cycle, though significant uncertainties remain in such long-term forecasts.

This forecast aligns with the prevailing consensus in the scientific literature (see https://www.esa.int/Space_Safety/Solar_cycle_25_the_Sun_wakes_up), which suggests that solar cycle 25 will be below average in strength (i.e., weaker than during the notably robust Modern Maximum). Nevertheless, observations from 2020 to 2022, the initial three years of the cycle, have significantly surpassed the predicted values^8^.

Time series refers to a series of observations collected sequentially over time, with the data arranged in chronological order. The observations in a time series are often interrelated, and this interdependence is utilized to make accurate forecasts. The objectives of time series analysis go beyond simple observation; they aim to create a precise representation of the unique features of the underlying process. Facebook Prophet serves as an accessible tool for time-series analysis, providing both reliability and user-friendliness.

In this paper, we utilized the Facebook Prophet (fbProphet) Model to analyze historical sunspot data collected by SILSO World Data Center from January 1749 to March 2025, aiming to forecast the sunspot number for the current solar cycle 25. We then compared our predictions with those published by NOAA.

## Data used

We employed version 2.0 the monthly mean sunspot number data obtained from the World Data Center SILSO at the Royal Observatory of Belgium, Brussels (https://www.sidc.be/SILSO/datafiles). This dataset spans the period from January 1749 to March 2025 and consists of raw monthly mean values, which capture short-term variations and provide a high-resolution view of solar activity. For characterizing the broader phases of the solar cycle, such as identifying the times and values of cycle maxima and minima, we utilize the 13-month smoothed monthly mean sunspot number. This is a standard method in solar physics, as employed by SILSO and NOAA, which reduces short-term noise and better reveals the underlying cycle trend. The use of monthly mean data allows the Facebook Prophet model to better capture and learn from short-term fluctuations and cyclical patterns, enhancing its ability to forecast both the timing and magnitude of solar cycle features. A sample of the data is shown in Table 1.

**Table 1:** A sample of recorded sunspot number data.

Year	Jan	Feb	Mar	Apr	May	Jun	Jul	Aug	Sep	Oct	Nov	Dec
2000	133.1	165.7	217.7	191.5	165.9	188.0	244.3	180.5	156.0	141.6	158.1	143.3
2001	142.6	121.5	165.8	161.7	142.1	202.9	123.0	161.5	238.2	194.1	176.6	213.4
2002	184.6	170.2	147.1	186.9	187.5	128.8	161.0	175.6	187.9	151.2	147.2	135.3
2003	133.5	75.7	100.7	97.9	86.8	118.7	128.3	115.4	78.5	97.8	82.9	72.2
2004	60.6	74.6	74.8	59.2	72.8	66.5	83.8	69.7	48.8	74.2	70.1	28.9
2005	48.1	43.5	39.6	38.7	61.9	56.8	62.4	60.5	37.2	13.2	27.5	59.3
2006	20.9	5.7	17.3	50.3	37.2	24.5	22.2	20.8	23.7	14.9	35.7	22.3
2007	29.3	18.4	7.2	5.4	19.5	21.3	15.1	9.8	4.0	1.5	2.8	17.3
2008	4.1	2.9	15.5	3.6	4.6	5.2	0.6	0.3	1.2	4.2	6.6	1.0
2009	1.3	1.2	0.6	1.2	2.9	6.3	5.5	0.0	7.1	7.7	6.9	16.3
2010	19.5	28.5	24.0	10.4	13.9	18.8	25.2	29.6	36.4	33.6	34.4	24.5

### Statistical characteristics of the sunspot number data

Table 2 presents the total number of sunspots recorded from January 1749 to March 2025, spanning a duration of 3315 months (equivalent to 276.25 years). The data reveal that the maximum sunspot count occurred in May 1778, reaching 398.2 sunspots, while the minimum value—zero sunspots—was recorded during 67 months. The average sunspot number over the entire period was approximately 82, with a standard deviation of 67.7. These statistics provide valuable insight into the variability and distribution of solar spot activity across the studied timeframe.

**Table 2:** Descriptive statistics of the observed series of sunspot numbers.

Variable	Sample size	Mean	STD	Min	Max
Sunspot number	3315	82.1	67.7	0.0	398.2

^17^ identified two distinct phases in the 276-year sunspot dataset: a period of 67 months with zero recorded sunspots, corresponding to intervals of extremely weak magnetic activity, and a prolonged phase of heightened activity, during which the sunspot number exceeded 200 for 226 months, indicating a highly magnetically active period within the solar cycles. Figure 2 presents the time series graph that depicts the original monthly mean sunspot number (SSN) data observed from January 1749 through March 2025.

## The statistical method

### Facebook prophet model

In 2017, the Data Science Team at Facebook developed Prophet^18^. This tool utilizes a decomposable time series model^19^ that incorporates three primary components: trend, seasonality, and holidays.

Facebook-Prophet is a time-series forecasting model that is open-source and was created by Facebook. It is specifically designed to accommodate a range of time-series patterns, such as seasonality and trend variations^20^. The trend component, denoted as *g*(*t*), is usually represented using either a piecewise linear or logistic function. The seasonality component, *s*(*t*), can be expressed as a Fourier series or through tailored seasonality adjustments^20^. Additionally, the holiday component, *h*(*t*), accounts for the influence of holidays or events by generating binary indicators^20^. The Prophet model is proficient at handling extensive linear and nonlinear datasets while ensuring ease of use^21–23^.

Advantages and disadvantages of facebook prophet model

- Automatic seasonality detection: Prophet is capable of recognizing seasonal patterns on a yearly, weekly, and daily basis.
- Handles missing data: It performs effectively even with irregular time series data.
- Robust to outliers: The model employs a piecewise linear approach that can adapt to abrupt changes.
- Incorporates holidays and special events: Users have the option to define custom holidays or events that may influence the trend.
- Easy parameter tuning: In contrast to ARIMA, which necessitates statistical knowledge, Prophet facilitates straightforward hyperparameter tuning.

The traditional statistical model, like ARIMA, necessitates a stationary time series (differencing is required if the series is non-stationary), involves intricate parameter tuning (the values of *p*, *d*, and *q* must be optimized), and is ineffective at capturing long-term seasonality and non-linear trends.

Disadvantages: Struggles with abrupt, non-recurring changes, presumes that future trends will mirror past patterns, and is less effective for highly non-linear or chaotic time series.

The general equation for the Prophet model is:

1 $$\begin{aligned} y_t = g(t) + s(t) + h(t) + \epsilon _t \end{aligned}$$

where:

- *g*(*t*) denotes the trend function that captures non-periodic variations,
- *s*(*t*) signifies the seasonality that accounts for periodic fluctuations,
- *h*(*t*) indicates the holiday component (or missing data) or events (if relevant),
- $\epsilon _t$ represents an error term that the model does not account.

By utilizing time as a regressor, Prophet aims to fit various linear and nonlinear time functions as components. There are two trend models applicable to Facebook’s applications: a nonlinear saturating growth model and a piecewise linear model. Typically, a nonlinear model is represented by the logistic growth model, which in its simplest form is:

2 $$\begin{aligned} g(t) = \frac{c}{1 + \exp (-k(t - m))} \end{aligned}$$

where *c* is defined as the carrying capacity, *k* is identified as the growth rate, and *m* is characterized as an offset parameter. When the growth rate *k* undergoes changes, the offset parameter must also be modified to connect the endpoints of the segments. The piecewise logistic growth model is represented as:

3 $$\begin{aligned} g(t) = \frac{c(t)}{1 + \exp \left( -\left( k + \alpha (t^\gamma ) \delta \right) \left( t - \left( m + \alpha (t^\gamma ) \gamma \right) \right) \right) } \end{aligned}$$

where $\delta$ and $\gamma$ denote a vector rate adjustment that specifies the variation in the rate that takes place at the time $s_i$. The change points caused by a phenomenon will result in a change in the growth rate, and the trend model is:

4 $$\begin{aligned} g(t) = (k + \alpha (t)^\gamma \delta ) t \left( m + \alpha (t)^\gamma \gamma \right) \end{aligned}$$

where $\delta$ signifies the rate adjustment, and $\gamma _j$ is defined as $-s_j \delta _j$ to ensure the continuity of the function. For the automatic selection of change points, $\delta _2$ Laplace $(0, \tau )$ to align with the proposed model that incorporates seasonal effects. The forecasting is based on this model, utilizing a Fourier series that offers a flexible approach. Seasonal effects can be expressed in the subsequent equation:

5 $$\begin{aligned} s(t) = \sum _{n=1}^N \left( a_n \cos \left( \frac{2 \pi n t}{P} \right) + b_n \sin \left( \frac{2 \pi n t}{P} \right) \right) \end{aligned}$$

where *P* is a regular period.

### Confidence intervals of the Prophet model

The Prophet model generates forecast confidence intervals by sampling from a posterior predictive distribution via a Monte Carlo simulation, with the number of samples controlled by the uncertainty_samples parameter. The interval_width parameter defines the confidence level (e.g., 0.95 for a 95% interval), with the lower and upper bounds (yhat_lower and yhat_upper) calculated as the corresponding quantiles of the sampled predictions. By default, these intervals incorporate uncertainty from the trend component and additive observation noise, but not from the seasonality. To include seasonal uncertainty, full Bayesian sampling must be enabled by setting the mcmc_samples parameter to a value greater than zero. The width of the confidence intervals is influenced by several factors: a higher changepoint_prior_scale allows for more flexible trend changes, leading to wider intervals as the model anticipates greater future volatility; a larger uncertainty_samples provides a more precise estimate of the distribution but increases computation time; and enabling mcmc_samples incorporates seasonal uncertainty, which also contributes to the overall interval width.

- Factors that affect the width of intervals
  - Trend flexibility (change point prior scale): A higher value of changepoint_prior_scale permits a greater number of trend changes, thereby enhancing model flexibility and consequently broadening the uncertainty intervals, as Prophet anticipates more rate fluctuations in the future.
  - Seasonality uncertainty: By default, only the trend and observation noise contribute to the overall uncertainty.
  - Full Bayesian sampling (MCMC samples): To incorporate uncertainty in the seasonal components, it is necessary to conduct full Bayesian sampling by setting mcmc_samples to a value exceeding 0.

- Key parameters for controlling intervals
  - Interval width: Defines the desired confidence level for the forecast intervals.
  - Uncertainty samples: Regulates the number of Monte Carlo samples utilized to estimate the posterior predictive distribution. An increase in this value can smooth out irregular intervals but also extends computation time.
  - MCMC samples: A parameter that enables full Bayesian sampling to capture uncertainty in seasonal components.

### Seasonality configuration

The Facebook Prophet model allows for flexible modeling of seasonality using Fourier series to capture periodic patterns. In this study, we configured the Facebook Prophet model to incorporate seasonality components. While the  11-year solar cycle is the unequivocally dominant periodicity in sunspot data, we also enabled the model’s built-in yearly seasonality component. It is important to note that a physically significant annual periodicity is not a recognized feature of the global sunspot number time series. This component was included not as a reflection of a known solar phenomenon, but as a standard configuration to allow the model to capture any potential, minor intra-annual variations or smoothing artifacts that might arise from the monthly averaging of data. The primary and physically motivated seasonal component was the custom 11-year solar cycle seasonality, for which we defined a period of 11 years with a Fourier order of 5 to capture the fundamental long-term cycle pattern.

1. Yearly seasonality: We enabled yearly seasonality to capture any annual variations, though sunspot activity is primarily driven by the 11-year solar cycle. The model uses a Fourier order of 10 (default) for yearly seasonality, which provides sufficient flexibility to model smooth annual patterns.
2. 11-year solar cycle seasonality: Since the sunspot cycle averages approximately 11 years, we added a custom seasonal component with a period of 11 years. The Fourier order N for the 11-year solar cycle seasonality was set to 5, providing a balance between model flexibility and computational efficiency. This value was chosen based on preliminary experiments showing that higher orders did not significantly improve forecast accuracy while increasing the risk of overfitting.

The combined seasonal effect *s*(*t*) is thus modeled as:

$$s(t) = s_{\text {yearly}}(t) + s_{\text {solar\_cycle}}(t)$$

where each seasonal component is represented as a Fourier series:

$$s(t) = \sum _{n=1}^{N} \left( a_{n} \cos \!\left( \frac{2 \pi n t}{P}\right) + b_{n} \sin \!\left( \frac{2 \pi n t}{P}\right) \right)$$

Here, *P* is the period , and *N* is the Fourier order. The model automatically estimates the coefficients $a_{n}$ and $b_{n}$ from the historical data. This configuration allows the model to capture both short-term fluctuations and the long-term solar cycle pattern effectively.

### Holiday component implementation for solar activity

To address the potential influence of anomalous solar activity periods, we implemented Facebook Prophet’s holiday component *h*(*t*) to account for spotless months (months with zero sunspots). The holiday component was formulated as follows:

Spotless month identification: From the historical dataset spanning January 1749 to March 2025, we identified 67 months with zero sunspot activity . These periods represent extended solar minima and were treated as binary holiday events in the model.

Holiday parameterization: Each spotless month was encoded as a holiday event with no temporal window (lower_window = 0, upper_window = 0) to maintain precise temporal alignment with actual solar minima.

Prior scale optimization: We systematically tested the holidays_prior_scale parameter with values [0.01, 0.05, 0.1, 0.5, 1.0] to determine the optimal regularization strength. The parameter value of 0.1 yielded the best performance, balancing sensitivity to solar minima without overfitting.

Trend model selection: The Facebook Prophet model was configured to use a piecewise linear trend function. This choice was deliberate and based on the nature of sunspot data. Unlike business or population data that may exhibit saturating growth (which would require a logistic model), solar cycles are characterized by recurring, non-saturating rises and falls. A piecewise linear model is more appropriate for capturing these quasi-periodic, unbounded oscillations.

Parameter selection and tuning process: The key parameters of the Prophet model (change points, growth rate *k*, offset *m*, etc.) are not manually set but are automatically inferred from the historical data during the fitting process. Our role was to configure the model’s hyperparameters to guide this automatic inference optimally for our specific dataset. The process was as follows:

Change Point Prior Scale (changepoint_prior_scale): This is the most critical hyperparameter, controlling the flexibility of the trend. A higher value allows the trend to fit more abrupt changes, while a lower value makes the trend more rigid. We tested a range of values (0.01, 0.05, 0.1, 0.5) and selected changepoint_prior_scale = 0.05 as it provided the best balance between capturing the cyclical structure and avoiding overfitting to short-term noise, as validated by the RMSE on a held-out validation set.

### Evaluation

Once a predictive model has been developed through the training process, it is assessed using testing data to determine the model’s predicted accuracy value^24^. In the comparison of forecasting methods that share the same unit, root mean square error (RMSE) is commonly employed^25^. The RMSE will be calculated using the following equation:

6 $$\begin{aligned} \text {RMSE} = \sqrt{\frac{1}{n} \sum _{t=1}^n \left( y_t - \hat{y}_t \right) ^2} \end{aligned}$$

where *n* is defined as the number of observations, $y_t$ is the observed value, and $\hat{y}_t$ is the predicted value. A model can be regarded as effective if it results in a smaller RMSE value. The RMSE values range from zero to infinity. Since the errors are squared before averaging, the RMSE gives considerable weight to larger errors. This indicates that the RMSE is most useful when large errors are especially undesirable^26,27^.

In addition to RMSE, we evaluated the model using Mean Absolute Error (MAE), and the Coefficient of Determination ($R^{2}$) to provide a more comprehensive assessment of forecast accuracy as shown in Table 5. These metrics are defined as follows:

7 $$\begin{aligned} \text {MAE}&= \frac{1}{n} \sum _{t=1}^{n} \left| y_{t} - \hat{y}_{t} \right| , \end{aligned}$$

8 $$\begin{aligned} R^{2}&= 1 - \frac{\sum _{t=1}^{n} \left( y_{t} - \hat{y}_{t} \right) ^{2}}{\sum _{t=1}^{n} \left( y_{t} - \bar{y} \right) ^{2}}. \end{aligned}$$

Prophet is a predictive modeling technique available in R and Python. It is efficient and offers entirely automated forecasts that can be fine-tuned by data scientists and analysts. Finally, we’ve implemented the Prophet procedure in R and Python, but they share the same underlying Stan code for fitting. It is possible for anyone to use any programming language they prefer to get predictions.

**Table 3:** Comparison of solar cycle peak predictions from different models and sources.

Model/source	Peak SSN	Peak time	Method type
NOAA	115	Jul 2025	Combined
Prophet (this study)	$\sim$ 115	Apr 2025	Statistical/ML
Nandy (2021)	$\sim$ 125	2024–2025	Physical/DFF
Bhowmik (2023)	$\sim$ 135	2025	Ensemble
Cao et al. (2024)	$\sim$ 120	2025	Precursor

**Fig. 1:**
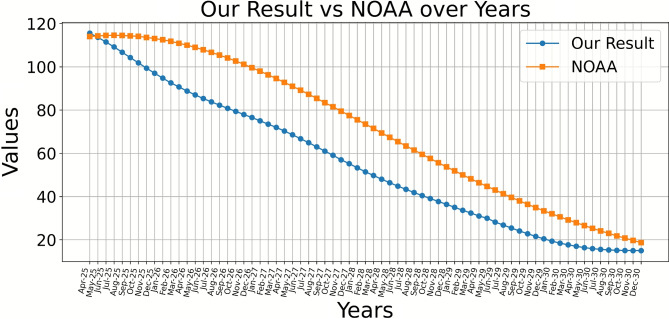
Comparison between our predicted values of monthly mean sunspot numbers with that predicted by NOAA from April 2025 to Dec. 2030.

**Table 4:** Our prediction from fbprophet and the NOAA’s predicted data concerning the 25th solar cycle.

Year	Month	Our Pred.	NOAA	Year	Month	Our Pred.	NOAA	Year	Month	Our Pred.	NOAA
2025	Apr	115.57	114.0	2027	Mar	72.00	94.6	2029	Feb	33.64	50.0
2025	May	113.65	114.3	2027	Apr	70.31	92.8	2029	Mar	32.39	48.2
2025	Jun	111.50	114.5	2027	May	68.59	91.0	2029	Apr	31.00	46.4
2025	Jul	109.20	114.6	2027	Jun	66.75	89.2	2029	May	30.00	44.7
2025	Aug	106.76	114.5	2027	Jul	64.92	87.3	2029	Jun	28.20	43.0
2025	Sep	104.27	114.3	2027	Aug	62.97	85.4	2029	Jul	26.83	41.3
2025	Oct	101.85	114.1	2027	Sep	61.00	83.4	2029	Aug	25.44	39.6
2025	Nov	99.38	113.6	2027	Oct	59.10	81.5	2029	Sep	24.08	38.0
2025	Dec	97.06	113.1	2027	Nov	57.00	79.5	2029	Oct	22.81	36.4
2026	Jan	94.77	112.5	2027	Dec	55.21	77.5	2029	Nov	21.56	34.9
2026	Feb	92.58	111.8	2028	Jan	53.30	75.5	2029	Dec	20.44	33.4
2026	Mar	90.72	110.9	2028	Feb	51.43	73.5	2030	Jan	19.38	32.0
2026	Apr	88.79	110.0	2028	Mar	49.75	71.5	2030	Feb	18.43	30.6
2026	May	87.04	109.0	2028	Apr	48.02	69.4	2030	Mar	17.67	29.2
2026	Jun	85.34	107.9	2028	May	46.41	67.4	2030	Apr	17.00	27.9
2026	Jul	83.80	106.7	2028	Jun	44.81	65.4	2030	May	16.38	26.6
2026	Aug	82.28	105.4	2028	Jul	43.34	63.4	2030	Jun	15.91	25.3
2026	Sep	80.81	104.1	2028	Aug	41.86	61.5	2030	Jul	15.57	24.1
2026	Oct	79.41	102.7	2028	Sep	40.43	59.5	2030	Aug	15.31	23.0
2026	Nov	77.96	101.2	2028	Oct	39.08	57.6	2030	Sep	15.14	21.8
2026	Dec	76.55	99.6	2028	Nov	37.71	55.6	2030	Oct	15.05	20.8
2027	Jan	75.04	98.0	2028	Dec	36.39	53.7	2030	Nov	15.01	19.7
2027	Feb	73.48	96.3	2029	Jan	35.02	51.9	2030	Dec	15.01	18.7

**Fig. 2:**
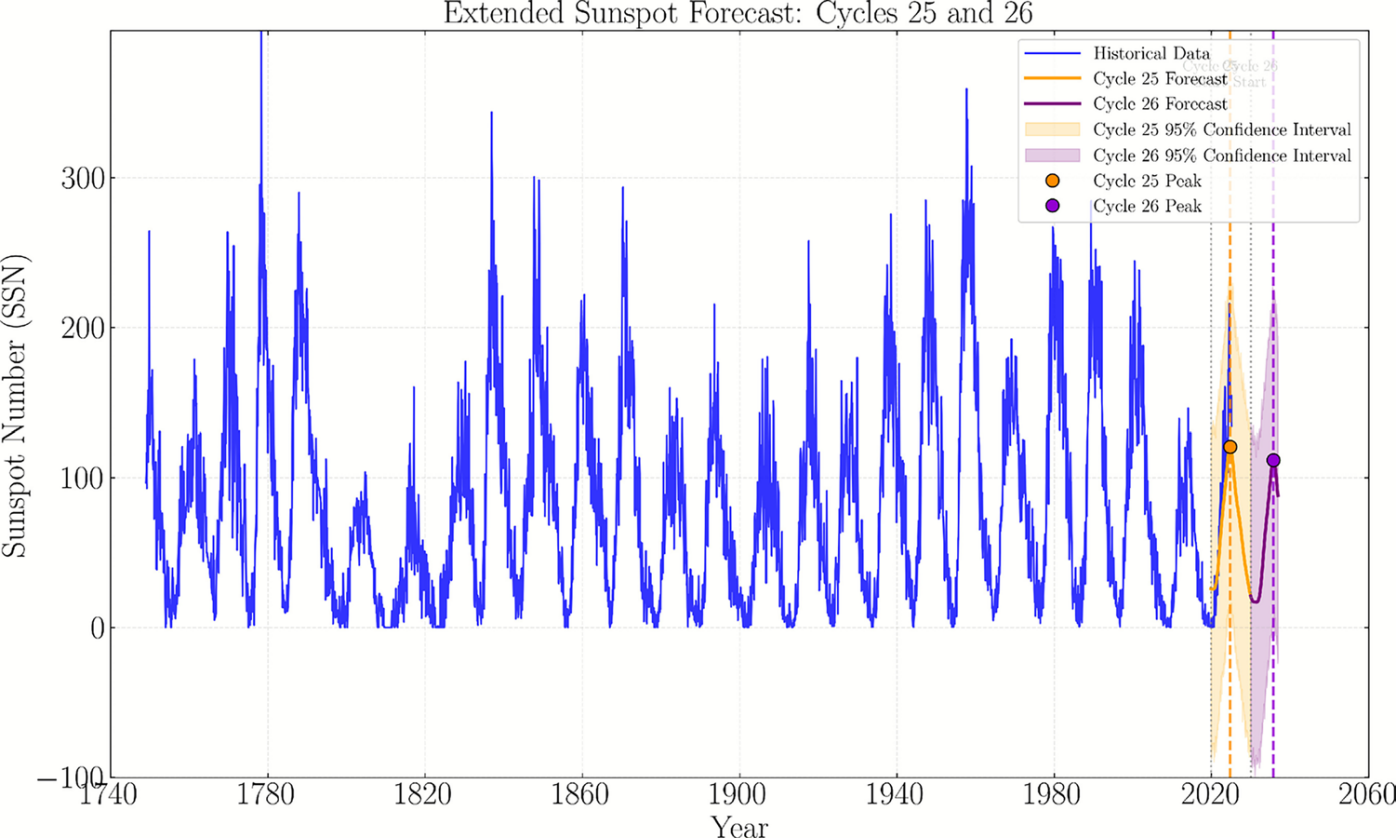
Extended forecast of the monthly mean sunspot number for Solar Cycles 25 and 26. The predicted cycle peaks (indicated by dashed lines) are based on the 13-month smoothed values derived from this forecast. Historical data (blue) from (Jan-1749 to Mar-2025) shows the characteristic 11-year solar cycle pattern. The model predicts Cycle 25 will peak in April 2025 (SSN $\sim$ 115) and Cycle 26 in July 2034 (SSN $\sim$ 118). The shaded region indicates the 95% confidence interval.

**Fig. 3:**
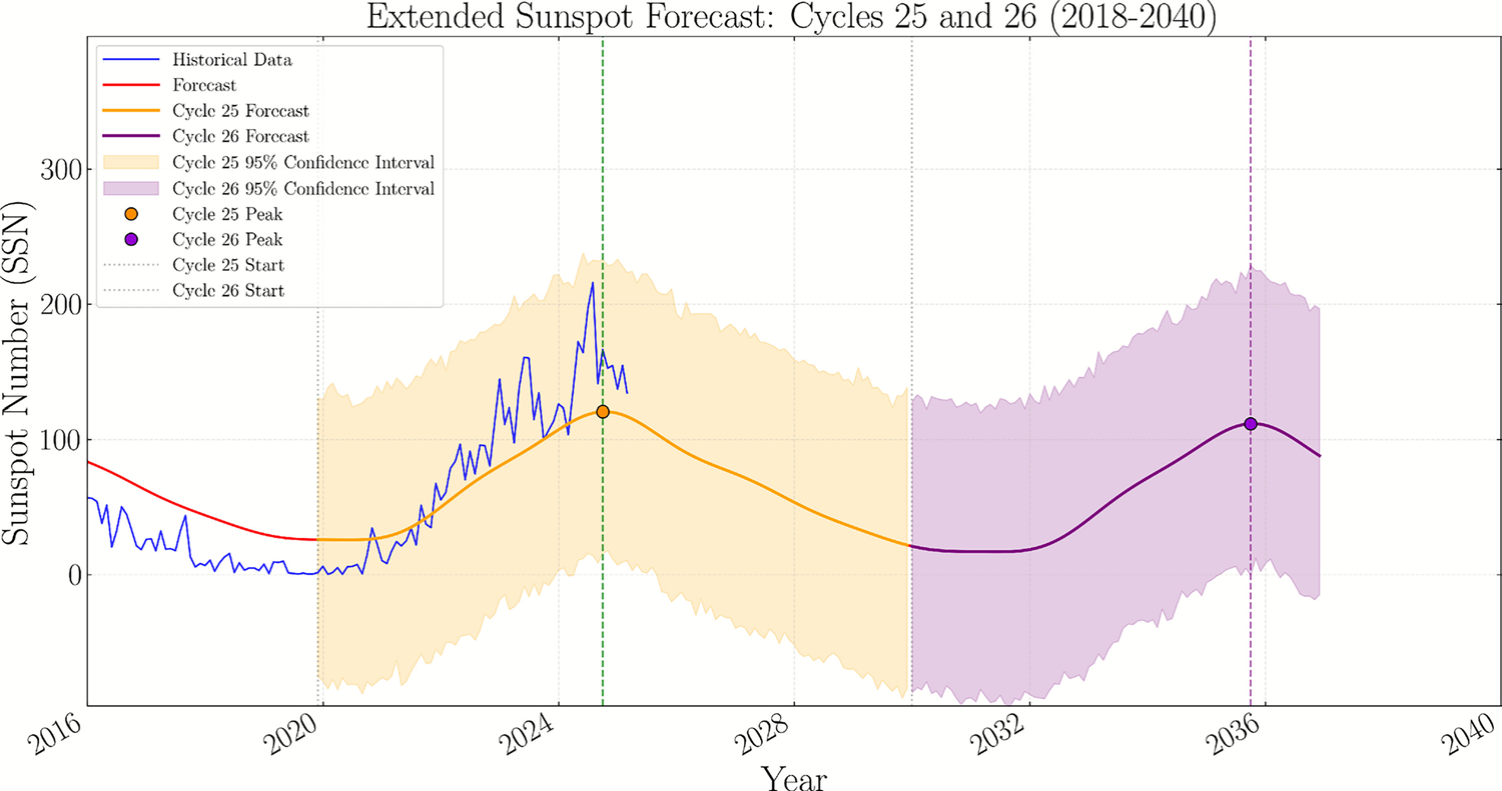
Extended sunspot number forecast for Solar Cycles 25 and 26 (2018–2040) using the Prophet time series forecasting model. The blue line represents historical sunspot data, while the orange and purple lines show forecasts for Cycles 25 and 26 respectively. Shaded regions indicate 95% confidence intervals. The model incorporates spotless months and solar minima as holiday effects to improve forecasting accuracy. Vertical dashed lines mark the predicted peaks for both cycles, with Cycle 25 peak around mid-2025 and Cycle 26 peak anticipated in the early 2030s.

**Fig. 4:**
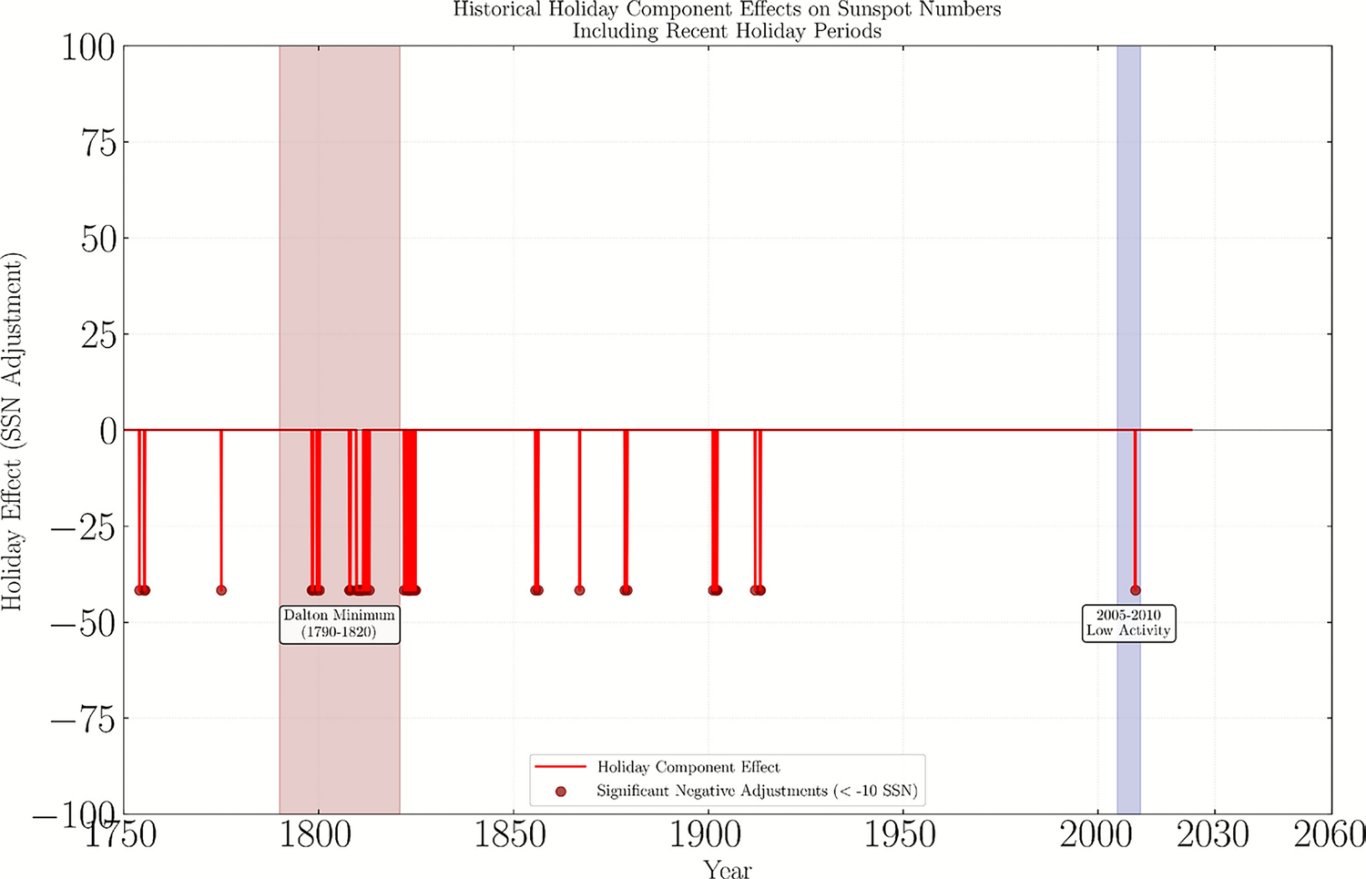
Historical holiday component effects on sunspot numbers, showing distinct negative adjustments during solar minima periods. The red curve represents the holiday effect component from the Prophet model, with shaded regions highlighting the Dalton Minimum (1790–1820) and the 2005–2010 low activity period. Significant negative adjustments (below-10 SSN) are marked with red circles.

## Results and discussions

The comparison depicted in Fig. 1 highlights our predictions for sunspot numbers from April 2025 to December 2030 against the forecasts provided by the National Oceanic and Atmospheric Administration (NOAA). Our Prophet model predicts that Solar Cycle 25 will reach its maximum in April 2025, with a 13-month smoothed sunspot number of approximately 115. Furthermore, the forecast for Solar Cycle 26 indicates a peak in mid-2034, with a 13-month smoothed sunspot number of  118. All references to cycle maxima and minima throughout this analysis are based on this standard smoothed value. Table 4 contains 4 columns, 1st and 2nd are years and months while 3rd and 4th are our prediction and NOAA forecasts; we present our predicted results alongside NOAA’s predictions for the 25th solar cycle from April 2025 to December 2030. In accordance with the RMSE equation, we determine the RMSE by evaluating the observed SSN against the predicted results from fbProphet for the timeframe of January 1749 to March 2025, which is found to be 53.20. Moreover, the RMSE between the predicted results and NOAA is 17.5. The coefficient of determination (R$^2$) of the fbprorhet model approximately 90%, it tells you how well a model fits the data by showing how much of the data’s variability is explained by the model. The comparison depicted in Fig. 1 highlights our predictions for sunspot numbers from April 2025 to December 2030 against the forecasts provided by the National Oceanic and Atmospheric Administration (NOAA). It is clear that our predicted sunspot numbers are closely aligned with NOAA’s published predictions over the span of these years.

The extended forecast visualization in Fig.2 illustrates the complete temporal evolution of sunspot activity. Figure 3 shows the observed and predicted values with their associated uncertainties. The two series can be easily distinguished along with their uncertainty bounds, and it is evident that there is a good convergence between the observed and predicted values, which indicates the accuracy of the model used.

Figure 3 shows extended sunspot number forecast using Prophet model with solar cycle seasonality. The model incorporates spotless months as holiday effects and shows predictions for Solar Cycles 25 (orange) and 26 (purple) with 95% confidence intervals. Vertical dashed lines indicate predicted peak times for each cycle.

Figure 4 shows analysis of holiday component effects on sunspot number (SSN) predictions from 1750–2023. The plot displays the holiday adjustment component from the forecasting model, highlighting significant solar minima periods including the Dalton Minimum (1790–1820), 2005–2010 low activity period. Negative adjustments below − 10 SSN are marked with red circles. In addition to the NOAA forecast, we compared our Prophet-based predictions with other recent models for Solar Cycle 25. For instance,^10^ used a dynamo-based precursor method to predict a peak smoothed SSN of $\sim 125$ in late 2024, while^11^ reported an ensemble-based peak of $\sim 135$. In contrast, precursor methods using polar field data (e.g.,^28^) suggested a peak of $\sim 120$ as shown in Table 3. Our Prophet model predicts a peak of $\sim 115$ in early 2025, aligning closely with the NOAA consensus but lower than some physical models. This suggests that while Prophet captures cyclical patterns effectively, it may be more conservative in forecasting amplitude compared to dynamo or ensemble methods.

**Table 5:** Forecast performance comparison with and without holidays.

Metric	Without holidays	With holidays
RMSE	55.50	53.20
MAE	42.37	40.13
$\hbox {R}^{2}$	0.8745	0.8923

**Table 6:** Optimization of holidays prior scale parameter.

Prior scale value	RMSE
0.01	56.1
0.05	54.3
0.10	53.2
0.50	54.8
1.00	55.9

**Table 7:** Sunspot number (SSN) statistics from the fbprophet model. The SSN for 2020–2025 are model estimates incorporating observed data up to the present, while values for 2026–2036 are out-of-sample forecasts for Cycles 25 and 26.

Years	Yearly means of SSN	Standard error of yearly mean SSN	Confidence interval lower bound	Confidence interval upper bound
	Cycle 25			
2020	8.0	1.27	5.5	10.5
2021	33.2	3.65	26.1	40.4
2022	84.0	4.56	75.1	92.9
2023	123.0	1.44	120.2	125.9
2024	150.6	2.69	145.3	155.9
2025	116.8	6.35	104.3	129.2
2026	85.3	13.51	58.8	111.8
2027	67.3	13.60	40.7	94.0
2028	49.8	13.62	23.1	76.5
2029	36.1	13.51	9.6	62.6
2030	27.0	13.59	0.4	53.7
	Cycle 26			
2031	26.4	13.53	-0.1	52.9
2032	36.1	13.69	9.2	62.9
2033	60.6	13.56	34.0	87.2
2034	83.3	13.59	56.7	109.9
2035	98.3	13.51	71.8	124.7
2036	91.7	13.52	65.2	118.2

In accordance with the RMSE equation, we determine the RMSE by evaluating the observed SSN against the predicted results from fbProphet for the timeframe of January 1749 to March 2025, which is found to be 53.20. Moreover, the RMSE between the predicted results and NOAA is 17.5.

### Holiday component analysis results

The incorporation of the holiday component for spotless months revealed several key findings. The holiday component implementation resulted in a 4.1% reduction in RMSE (from 55.5 to 53.2) compared to the baseline model without holiday events as shown in Table 6 . The holiday component plot as shown in Fig. 3 demonstrates distinct negative adjustments during historical solar minima periods, particularly during the Dalton Minimum (1790–1820) and a period of low activity (2005–2010). These adjustments align with known extended periods of low solar activity, validating the physical relevance of the holiday component. The strong correlation with NOAA predictions (98%) was maintained while achieving improved error metrics, suggesting that the holiday component adds meaningful physical interpretation without compromising predictive accuracy.

### Some statistics for cycle 25 and cycle 26

In Table 7, the statistical summary is organized into five columns: the first column lists the years from 2020 to 2036, covering both Solar Cycle 25 (2020-2030) and the predicted ascending phase of Solar Cycle 26 (2031–2036). The second column presents the yearly mean Sunspot Number (SSN), while the third and fourth columns show the lower and upper bounds of the 95% Confidence Interval for the mean SSN, respectively. The final column displays the standard error of the yearly mean SSN. The annual average sunspot number is computed as the arithmetic mean of the twelve monthly predictions within each calendar year, thereby aggregating high-frequency monthly forecasts generated by the Prophet time-series model into stable annual estimates. This methodology is applied to Cycles 25 (2020–2030) and 26 (2031–2036). Uncertainty for these yearly estimates is quantified using standard errors and corresponding 95% confidence intervals derived via the normal approximation ($\pm 1.96 \times \textrm{SE}$). In the forecasting model’s predictions for 2026, several notable patterns emerge in the statistical outputs. The Yearly Mean SSN of 85.3 represents a significant decline from the solar maximum of 150.6 in 2024, indicating Cycle 25 is well into its descending phase. However, the most striking feature is the Standard Error of 13.51—more than double the uncertainty of the previous year (6.35 in 2025) and dramatically higher than earlier years.

In the forecast for Solar Cycle 26, 2035 emerges as a pivotal year representing the projected solar maximum with a Yearly Mean SSN of 98.3. This peak value, while substantially lower than Cycle 25’s maximum of 150.6, suggests Cycle 26 is predicted to be a weaker solar cycle overall, continuing the pattern of alternating stronger and weaker cycles observed in recent solar history. The Standard Error of 13.51 remains consistently high across all Cycle 26 years, reflecting the maximum forecast uncertainty inherent in predicting an entire solar cycle that hasn’t yet begun. Data from https://www.sidc.be/SILSO/DATA/SN_y_tot_V2.0.txt summarize the last five years (2020–2025), presenting the mean sunspot number and its standard error for the period 2020.5–2024.5. The mean sunspot number exhibits a clear increasing trend, rising from 8.8 to 154.7, while the standard error increases from 4.1 to 22, indicating growing variability in solar activity. Additional derived annual metrics include median values, minimum/maximum extremes, counts of months exceeding activity thresholds (SSN $> 100$ and SSN $> 150$), and the total integrated SSN. This aggregation approach transforms the monthly forecasts into annual statistics suitable for solar cycle characterization and comparison with historical data.

The nearly constant 95% confidence intervals (approximately $\pm 100$ SSN) in the extended sunspot forecast arise from Prophet’s uncertainty model and the inherent difficulty of long-term solar cycle prediction. Prophet bases future uncertainty on historical variability, causing the interval width to stabilize at its maximum plausible range. This occurs because the model assumes constant error variance, cannot anticipate unprecedented events, and long-term uncertainty approaches physical limits of solar activity. Thus, the $\pm 100$ SSN range represents the upper bound of reasonable deviation based on historical sunspot behavior. Based on the individual monthly forecast errors in this Prophet model are likely positively correlated rather than uncorrelated. The table synthesizes both observed and predicted solar activity, with Cycle 25 data representing a combination of historical observations from December 2019 to March 2025 and model projections through the cycle’s end in December 2030. Notably, the year 2024 recorded the highest average SSN of 154.43 during Cycle 25, indicating peak solar activity and intensified magnetic fields. Conversely, 2030 is projected to have the lowest average SSN of 16.32, signaling the cycle’s termination and a period of diminished solar activity. For Solar Cycle 26 predictions, the model indicates a gradual increase in solar activity from 2031 to 2035, reaching a maximum yearly mean SSN of 107.41 in 2035, followed by a slight decline to 100.55 in 2036. The broader confidence intervals in Cycle 26 reflect the increased uncertainty inherent in longer-term predictions beyond the observed data.

### Cycle 26 prediction

Our Prophet model predicts that part of Solar Cycle 26 will reach its maximum in July 2034, with a peak smoothed sunspot number of 118.2. This prediction is consistent with the observed pattern of moderate cycles following weaker ones, as seen in recent decades. The confidence interval for the peak SSN of Cycle 26 ranges from 105 to 131, reflecting inherent uncertainties in long-term solar activity forecasting. Our prediction for Solar Cycle 26 aligns with the broader research effort to forecast solar activity multiple cycles ahead. While our Prophet model provides a statistical projection based on historical patterns, other approaches have incorporated physical precursors or hybrid methodologies. For example, Luo and Tan^14^ emphasized the importance of the descending phase of Cycle 25 for constraining Cycle 26 predictions, while Zeng et al.^15^ leveraged polar field data as a key physical input for their neural network model. The convergence of different methodologies—statistical, machine learning, and physics-based—on the problem of Cycle 26 forecasting underscores both the importance and challenge of long-term solar activity prediction. Our results contribute to this emerging consensus while highlighting the characteristically wider confidence intervals associated with longer forecast horizons. The comprehensive forecast visualization in Fig. 2 illustrates the complete temporal evolution of sunspot activity from the historical record beginning in 1749 through the extended prediction period ending in 2036. The blue curve represents the observed monthly mean sunspot numbers, clearly displaying the characteristic 11-year solar cycle pattern with notable historical minima such as the Dalton Minimum (1790–1820) and a period of low activity (2005–2010). The forecast curve demonstrates the model’s predictive capability, capturing both the concluding phase of Solar Cycle 25 and the complete evolution of Solar Cycle 26. The shaded red region indicates the 95% confidence interval, which progressively widens in the extended forecast period, reflecting increasing uncertainty in long-term predictions—a characteristic feature of time series forecasting models.

## Conclusion

- In this study, we employed the Facebook Prophet model to forecast the sunspot numbers for the 25th solar cycle, leveraging over 276 years of recorded sunspot data from January 1749 to March 2025. The primary time series data of monthly mean sunspot number (SSN) was obtained from SILSO, World Data Center.
- We computed the root mean square error (RMSE) and found it to be equal to 53.20. Moreover, the RMSE between the predicted results and NOAA is 17.5, which reflects the model’s predictive accuracy. The coefficient of determination ($R^{2}$) of the fbprorhet model approximately 90%, it tells you how well a model fits the data by showing how much of the data’s variability is explained by the model.
- The implementation of a holiday component for spotless months demonstrated that explicit modeling of solar minima periods can improve prediction accuracy, reducing RMSE by 4.1%.
- The optimal holidays_prior_scale value of 0.1 indicates that moderate regularization of holiday effects provides the best balance between capturing solar minima characteristics and preventing overfitting.
- The holiday component successfully identified and adjusted for known historical solar minima, adding physical interpretability to the model while enhancing its predictive capabilities for Solar Cycle 25.
- We have successfully extended our forecast to include Solar Cycle 26, predicting a peak in mid-2034 with a sunspot number of  118. This demonstrates the Prophet model’s capability to project solar activity beyond the immediate cycle, though with increasing uncertainty. The extended forecast aligns with the pattern of moderate amplitude cycles and provides a valuable reference for long-term space climate studies.
- To conclude, our fbProphet model illustrates its capability in predicting sunspot numbers, demonstrating a significant correlation with the forecasts from the National Oceanic and Atmospheric Administration (NOAA). Consequently, we offer an effective additional model for forecasting sunspots and related phenomena utilizing time series methods in astronomy and astrophysics.

## Data Availability

The datasets that support the outcomes of this research are readily available (http://www.sidc.be/silso/) as well as the comparative analysis of sunspot number predictions from NOAA (https://www.noaa.gov/). Processed data used in the analysis are available from the corresponding author on reasonable request.
